# Islands of Langerhans

**Published:** 1913-11

**Authors:** 


Islands of Langerhans. D. P. Grimes, *Arch. des Sciences Biol.,* No. 1, 1912, perpetrates a gross heterodoxy, claiming that the insulæ are not morphologically distinct from the pancreatic lobules, but represent identical epithelial structures, either in a special functional condition or even undergoing retrograde metamorphosis. He even questions the existence of an internal secretion and asserts that if it does exist, it is formed both by the insulæ and the ordinary lobules. Without accepting this heresy, it is only fair to note that it would explain the marked failure of therapy of diabetes based on the accepted theory. Moreover, generally speaking, a long dispute between clinical observation and scientific theory has usually resulted in corroboration of the former, certain contributary factors having been overlooked in the preliminary scientific demonstrations. We cannot refrain from prophesying that much light will be shed on the pathogeny of diabetes when the long accepted physiology of the hepatic function of glycogenesis is elaborated from the pathologic side.

